# Advanced Intestinal Cancers often Maintain a Multi-Ancestral Architecture

**DOI:** 10.1371/journal.pone.0150170

**Published:** 2016-02-26

**Authors:** Christopher D. Zahm, Joseph M. Szulczewski, Alyssa A. Leystra, Terrah J. Paul Olson, Linda Clipson, Dawn M. Albrecht, Malisa Middlebrooks, Andrew T. Thliveris, Kristina A. Matkowskyj, Mary Kay Washington, Michael A. Newton, Kevin W. Eliceiri, Richard B. Halberg

**Affiliations:** 1 Department of Oncology, University of Wisconsin–Madison, Madison, Wisconsin, United States of America; 2 Laboratory for Optical and Computational Instrumentation (LOCI), University of Wisconsin–Madison, Madison, Wisconsin, United States of America; 3 Laboratory of Cell and Molecular Biology, University of Wisconsin–Madison, Madison, Wisconsin, United States of America; 4 Department of Surgery, University of Wisconsin–Madison, Madison, Wisconsin, United States of America; 5 Division of Gastroenterology and Hepatology, Department of Medicine, University of Wisconsin–Madison, Madison, Wisconsin, United States of America; 6 Department of Ophthalmology and Visual Sciences, University of Wisconsin–Madison, Madison, Wisconsin, United States of America; 7 Department of Pathology and Laboratory Medicine, University of Wisconsin–Madison, Madison, Wisconsin, United States of America; 8 Carbone Cancer Center, University of Wisconsin–Madison, Madison, Wisconsin, United States of America; 9 Department of Pathology, Microbiology and Immunology, Vanderbilt University School of Medicine, Nashville, Tennessee, United States of America; 10 Department of Biostatistics and Medical Informatics and Department of Statistics, University of Wisconsin–Madison, Madison, Wisconsin, United States of America; The University of Texas MD Anderson Cancer Center, UNITED STATES

## Abstract

A widely accepted paradigm in the field of cancer biology is that solid tumors are uni-ancestral being derived from a single founder and its descendants. However, data have been steadily accruing that indicate early tumors in mice and humans can have a multi-ancestral origin in which an initiated primogenitor facilitates the transformation of neighboring co-genitors. We developed a new mouse model that permits the determination of clonal architecture of intestinal tumors *in vivo* and *ex vivo*, have validated this model, and then used it to assess the clonal architecture of adenomas, intramucosal carcinomas, and invasive adenocarcinomas of the intestine. The percentage of multi-ancestral tumors did not significantly change as tumors progressed from adenomas with low-grade dysplasia [40/65 (62%)], to adenomas with high-grade dysplasia [21/37 (57%)], to intramucosal carcinomas [10/23 (43%]), to invasive adenocarcinomas [13/19 (68%)], indicating that the clone arising from the primogenitor continues to coexist with clones arising from co-genitors. Moreover, neoplastic cells from distinct clones within a multi-ancestral adenocarcinoma have even been observed to simultaneously invade into the underlying musculature [2/15 (13%)]. Thus, intratumoral heterogeneity arising early in tumor formation persists throughout tumorigenesis.

## Introduction

The heterogeneity of tumors has been recognized for decades. Neoplastic cells within a single tumor vary in several distinguishable properties including differentiation state, proliferation rate, metastatic potential, and therapeutic response. Historically, two models to explain intratumoral heterogeneity were proposed. The clonal evolution model asserts that different subclones arise from a single progenitor as a consequence of molecular changes followed by selection for dissimilar microenvironments within a tumor [1]. In contrast, the cancer stem cell model contends that a small population of stem cells originating from a single progenitor is responsible for tumor maintenance but the progeny can differentiate in several diverse ways [1]. In both models, two key assumptions are that tumors are uni-ancestral, derived from a single founder, and that a subclone will eventually accumulate mutations that drive metastasis to other organs.

Data have been steadily accruing for a third model of intratumoral heterogeneity: solid tumors can have a multi-ancestral architecture [2]. We have demonstrated that hereditary and carcinogen-induced intestinal tumors in the laboratory mouse can be derived from multiple founders [3, 4]. Our findings are consistent with those of another laboratory demonstrating that hereditary and sporadic colorectal tumors in humans can be multi-ancestral in architecture [5, 6]. Thus, contrary to a widely accepted paradigm in the field of cancer biology, solid tumors can have a multi-ancestral origin and consequently be heterogeneous even as they just begin to form.

The initial studies with animal models primarily relied on aggregation chimeras. Such mice were generated by fusing together early embryos. Typically, one embryo carried the Rosa26 lineage marker that expresses β-galactosidase so that cells from this embryo stain blue in the presence of 5-bromo-4-chloro-3-indolyl-β-D-galactopyranoside, whereas cells from the other embryo remain white. Tumors that are heterotypic, composed of blue and white neoplastic cells, have a multi-ancestral origin. The use of aggregation chimeras has provided novel insights into the early stages of tumorigenesis. Unfortunately, this powerful experimental tool has some significant limitations. The generation of aggregation chimeras is very time consuming and expensive, so the number of animals analyzed is often low. The clonal architecture of any tumor can be determined only following a lengthy staining procedure that includes two fixation steps and an overnight incubation at 37°C. The staining is often incomplete since the substrate must diffuse into the tumor; consequently not all tumors can be scored. Moreover, the harsh conditions prohibit any subsequent molecular analysis.

In this study, we developed a new mouse model that allows the clonal architecture of intestinal tumors to be determined *in vivo* and *ex vivo*, validated this model, and then used it to determine the clonal architecture of intestinal cancers. The results support the concept that adenomas have a multi-ancestral origin and that this early diversity persists as tumors transition from a benign to malignant state.

## Methods

### Mouse strains and generation of mosaic mice

Animal studies were conducted under protocols approved by the Institutional Animal Care and Use Committee at the University of Wisconsin in compliance with policies set by the Office of Laboratory Animal Welfare at the National Institutes of Health. C57BL/6J (B6), FVB/NJ (FVB), B6.Apc^Min^/J mice that were heterozygous for the *Min* allele of the *Adenomatous polyposis coli gene* (*Apc*^Min/+^), and B6.129(Cg)-Gt(ROSA)26Sor^tm4(ACTB-tdTomato,-EGFP)Luo^/J mice that were hemizygous for the mT/mG reporter were obtained from The Jackson Laboratory. FVB/N-Tg^(Fabp1-Cre)1^Jig mice hemizygous for the Fabp1-Cre transgene in which the promoter from the rat fatty acid binding protein is fused to the gene encoding Cre recombinase, resulting in mosaic expression of Cre prior to the completion of intestinal morphogenesis, were obtained from the National Cancer Institute Mouse Repository at Frederick. Stocks of these strains were maintained by sibling crosses and backcrossing with B6 or FVB mice that were imported every fifth generation. Offspring were genotyped using DNA isolated from toe clips for PCR as described by the suppliers of animals.

A mouse carrying the *Min* allele of *Apc* was identified in the laboratory of Dr. William F. Dove at the University of Wisconsin following mutagenesis with ethylnitrosourea [7, 8]. The founder and its progeny presented with pale feet owing to blood loss from tumors in the intestine [7]. Tumor multiplicity, growth, and progression in *Apc*^Min/+^ mice are dependent on genetic background. B6 *Apc*^Min/+^ mice develop on average 100 intestinal tumors and typically die by 150 days of age [7]. Tumors are initiated following the loss of APC activity in intestinal epithelial cells soon after birth and they are almost always benign adenomas [9–13]. By contrast, (SWRxB6) F1 *Apc*^Min/+^ hybrids develop few tumors and typically live a year or more [14]. The tumors in these mice are often invasive cancers that occasionally metastasize to regional lymph nodes, presumably owing to the increased lifespan [14].

Experimental mice were generated by first crossing B6 *mT/mG* females to B6 *Apc*^Min/+^ males to generate B6 *Apc*^Min/+^; *mT/mG* mice that were then crossed to FVB *Fabp1-Cre* mice to generate (FVBxB6)F1 *Apc*^Min/+^; *Fabp1-Cre*; *mT/mG* hybrids. Experimental F1 hybrids were allowed to age to 150 days or until moribund to assess the clonal architecture of tumors at different stages.

### Fluorescence endoscopy

Fluorescence endoscopy was performed using a modified Karl Storz Coloview system as described previously [15, 16]. Briefly, mice were anesthetized with isoflurane and given an enema of warm PBS to clear any fecal material. The endoscope was inserted approximately four centimeters and then slowly withdrawn using an Optotronics diode-pumped solid-state laser set to 437nm at a power of 100mW as the light source. Video was collected during the entire procedure and still images were captured of each tumor. Mice were allowed to wake up after the procedure.

Videos and still images were processed with the Fiji package of ImageJ to visualize normal and dysplastic crypts expressing green fluorescent protein.

### Imaging of whole mounts

The intestinal tracts were removed intact at necropsy, opened longitudinally, rinsed with PBS, fixed in 4% paraformaldehyde for 4 hours and stored at 4°C in 70% ethanol. The small intestine, cecum, and colon were mounted in 0.4% agarose in PBS and imaged to capture red fluorescence (mT; tdTomato) and green fluorescence (mG; EGFP) using either a Zeiss microscope at 12.5x and or Nikon microscope at 20x magnification (see S1 File for additional details).

Whole-mount images used for statistical analysis were obtained on the Zeiss microscope and processed using the Fiji image-processing package of ImageJ [17]. A macro was used to ensure consistency among images. Briefly, the macro searched a directory for Zeiss image files (ZVI) and converted them to Open Microscopy Environment’s (OME) tagged image file format (TIFF) using the LOCI Bio-Formats Importer/Exporter to preserve meta data [18]. It then completed the following set of functions with settings based on which protein was imaged (tdTomato or EGFP): subtracted background, enhanced local contrast (CLAHE), set autothreshold, and converted to binary. Once in binary, the areas of the image containing no tissue were filled with yellow. The result was a binary image in which white represents tissue expressing tdTomato and black represents tissue expressing EGFP (S1 Fig).

### Patch size analysis

To assess the relationship between mosaicism and clonal architecture, red and green patches of intestinal epithelium in experimental mice were analyzed using distance maps as previously described for aggregation chimeras [3, 4]. Briefly, flat binary images of each intestinal section were converted to distance map images, which indicate for each position the Euclidean distance to the nearest opposite color, using the EBImage software package [19]. The average minimum distance to the opposite color measures the mosaic patch size, which was compared to patch sizes from aggregation chimeras (S2 Fig).

### 3D reconstruction of tumors

Some adenomas from mice euthanized at 150 days of age were excised and optically cleared using 2,2-thiodiethanol (TDE) as previously described [20]. Cleared adenomas were mounted *en bloc* in 97% TDE and multiphoton excitation imaging was performed on a Prairie Technologies Ultima IV Imaging system at LOCI.

Image stacks from the Ultima IV were stitched together using the default settings in the Grid/Collection Stitching plug-in for ImageJ that is distributed as part of the Fiji project [21]. Background subtraction was performed on both channels using the ImageJ subtract background function with a rolling ball radius of 40 pixels. When showing EGFP only, a threshold was used to eliminate tissue autofluorescence. The threshold was applied equally to all areas of all images that were used in the reconstruction. Images were then opened using the ImageJ 3D viewer plug-in (Display as: Volume, Color: None; Threshold: 0, Resampling factor: 1) to generate high quality 3D renderings of the OME dataset.

### Immunohistochemistry

All excised tumors were either embedded in FSC 22 (Surgipath) and flash frozen or placed in formalin, processed and embedded in paraffin. Blocks were serially sectioned and arrayed as two 5μm sections per slide. Every tenth slide was stained with hematoxylin and eosin. For more complete analysis, slides of interest were stained by immunohistochemistry for CTNNB1 (1:200 Purified Mouse Anti-β-catenin Antibody, BD Transduction Laboratories) using the HistoMouse Max Kit (Invitrogen) or by immunofluorescence for green fluorescent protein (EGFP) or red fluorescent protein (tdTomato) (primary– 1:1000 Living Colors EGFP Monoclonal Antibody, Clontech, and secondary– 1:1000 goat anti-mouse Alexa Fluor 488, Invitrogen; and/or primary– 1:200 anti-tdTomato, Rockland and secondary– 1:1000 goat anti-rabbit Alexa Fluor 568, Invitrogen) and 4',6-diamidino-2-phenylindole (DAPI) by applying ProLong Gold with DAPI mounting media (Molecular Probes). Note that staining for EGFP and tdTomato was necessary because fixation quenches the fluorescent signal.

### Immunofluorescence

Histological sections stained by immunofluorescence were imaged as described in the whole-mount imaging section; the only change was the use of 4x, 5x, 10x or 20x objective lenses. Post-processing of imaging was not necessary. Histological sections stained by immunohistochemistry or hematoxylin and eosin (H&E) were imaged in the same manner with a halogen light source and no color filters.

### Pathology

The pathology of each lesion was assessed by two board-certified pathologists (KAM and MKW) by examining H&E stained slides. Tumors were classified as either adenoma with low-grade dysplasia, adenoma with high-grade dysplasia, intramucosal carcinoma, or invasive adenocarcinoma. The histologic criteria used for the classification of lesions are defined as follows: similar to that observed in human tubular adenomas, lesions with low-grade dysplasia exhibited hyperchromatic, elongated nuclei with stratification of nuclei half-way toward the luminal aspect with scatted mitosis. In high-grade dysplasia, the hyperchromatic nuclei are larger, rounded and often contain a prominent nucleolus, exhibit nuclear stratification to the luminal surface, demonstrate complex architecture with back-to-back or cribriform glands, and contain abundant mitotic figures (sometimes atypical) with areas of cell necrosis. In tumors exhibiting intramucosal carcinoma, the nuclear and cytologic features are identical to that seen with high-grade dysplasia, however these lesions also demonstrate neoplastic cell invasion beyond the basement membrane into the lamina propria or muscularis mucosa and can be associated with a desmoplastic reaction. For lesions to be classified as invasive adenocarcinomas, the neoplastic cells must invade beyond the muscularis mucosa into at least the submucosa. The histology of invasive tumors can vary such that tumors can be composed of neoplastic cells with well-formed glands or sheets of cells without gland formation, however in all instances there is an associated desmoplastic response.

### Sequencing of *Apc*

Paraffin sections were prepared for laser capture microdissection (LCM) of regions of interest by staining for EGFP using the HistoMouse Max Kit (Invitrogen) (primary– 1:1000 Living Colors EGFP Monoclonal Antibody, Clontech). Adjacent sections were then cut onto membrane slides and stained with methylene blue. Regions of interest were removed via LCM with the assistance of the Translational Research Initiatives in Pathology (TRIP) Lab, University of Wisconsin–Madison. Alternately, if the patches were large, tissue was scraped by hand from unstained slides containing formalin-fixed, paraffin-embedded sections. Genomic DNA was extracted from the isolated tissue using the Maxwell 16 automated purification system with Maxwell 16 FFPE Plus LEV DNA Purification Kit following the instructions of the manufacturer (Promega, Madison, WI). PCR primers were designed to amplify two regions of exon 15 (codons 747–953 and 1285–1513; S1 Table) of the mouse *Apc* gene, since most mutations occur in these two regions [8, 22]. PCR was conducted for 35 cycles. Each cycle consisted of 30 seconds at 94°C, 30 seconds at 55°C, and 1 minute at 72°C. All amplification procedures included DNA-free controls consisting of the amplification cocktail and nuclease-free water (Promega) in place of template DNA. PCR primers were then used for sequencing. Mutation discovery and analysis was conducted using the mSeq 6.0.1 (Norman Drinkwater, McArdle Laboratory for Cancer Research, Department of Oncology, School of Medicine and Public Health, University of Wisconsin–Madison) and Mutation Surveyor (Softgenetics) programs.

## Results

To study the clonal architecture of intestinal tumors, we generated mice carrying three genetic elements. The first two were transgenes that are necessary to establish a pattern of mosaicism in the intestine. One transgene consists of the rat fatty acid binding protein 1 promoter fused to the gene encoding Cre recombinase and the other has the *mT/mG* reporter. In cells that failed to express Cre, red fluorescent protein (tdTomato) was exclusively produced from the reporter even during intestinal tumorigenesis; in cells expressing Cre beginning at day E13.5 (prior to the completion of intestinal morphogenesis and before Min tumor formation), green fluorescent protein (EGFP) was exclusively produced as the gene encoding the tdTomato was deleted following recombination. Thus, these two elements created a variegated pattern of cells expressing red or green fluorescent proteins in the distal small intestine, cecum and colon (S3 Fig). This pattern was stable at all time-points assessed (S5 Fig). The third element was a single copy of the *Min* allele of the *Adenomatous polyposis coli* (*Apc*) gene [8]. This mutation predisposed the mice to the spontaneous development of tumors throughout the small intestine and colon. Since the reporter status is determined prior to tumors forming and Cre expression is stable during tumorigenesis, tumors consisting of a mixture of red and green neoplastic cells have a multi-ancestral architecture.

To characterize intestinal tumorigenesis in this model, a cohort of 20 mice was generated and euthanized at 150 days of age. The incidence of intestinal tumors was 100% with an average multiplicity of 11 ± 5. A low tumor burden was expected owing to the homogeneous F1 genetic background and was highly desirable as it limited the possibility of two independently initiated tumors coalescing as a consequence of proximity [23]. All tumors from this group of mice that were analyzed were adenomas with low-grade dysplasia.

**Fig 1:**
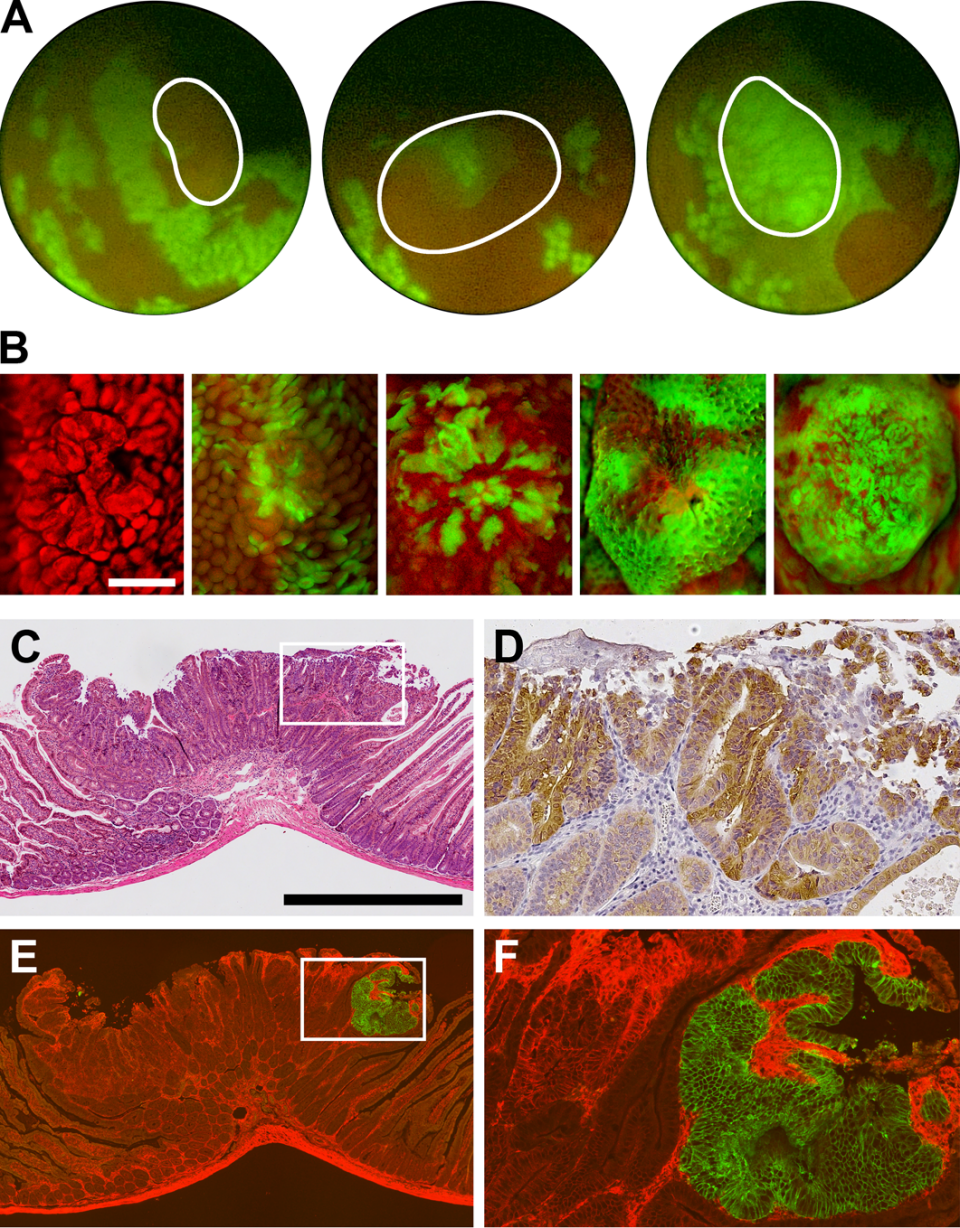
Mosaics are a powerful experimental tool for studying tumorigenesis. The clonal architecture of colon tumors is visible *in vivo* using fluorescence endoscopy. The intestinal epithelium is a single layer of cells that is replaced every three to five days. New cells are produced from stem cells lying near the base of the each crypt. In our model, crypts are homotypic red or homotypic green with the latter being particularly evident during fluorescence endoscopy, as each crypt appears as a discrete green circle (A). Tumors can be composed of only red crypts (left panel), a mixture of red and green crypts (middle panel), or only green crypts (right panel). The tumors are outlined in white. The high variability in clonal architecture is also evident in whole mounts (B). Tumors were homotypic red (left panel), heterotypic (middle three panels), or homotypic green (right panel). The mixture of red and green crypts varies among multi-ancestral tumors, with some being primarily composed of either red crypts or green crypts, whereas others are roughly an equal number of both types. Histology is necessary to determine architecture, as tumors can appear heterotypic with neoplastic crypts of one color intermingled with normal crypts of the other color. The clonal architecture of tumors is scored from histologic sections. Neoplastic cells were visualized following H&E staining (C) with high levels of CTNNB1 (β-catenin) expression in the cytoplasm and nucleus (D, brown stain). As shown in a nearby section from the same tumor stained by immunofluorescence, neoplastic cells within a single tumor often arose from both the red and green cell lineages (E-F). Panel B photos are shown at the same magnification; size bar = 1mm. Panels C and E are shown at the same magnification; size bar = 500μm. Panel D shows a 4x enlargement in an adjacent section of the outlined area in Panel C. Panel F shows a 4x enlargement of the area outlined in Panel E.

Many of the adenomas in these mice had a multi-ancestral architecture. The composition of colon tumors was evident *in vivo* by fluorescence endoscopy (Fig 1A) and *ex vivo* using fluorescent microscopy (Fig 1B). Of the 63 tumors in the distal small intestine and colon, 32 (51%) were confirmed to be composed of red and green neoplastic cells by histology (Table 1 and Fig 1C–1F). Features of transformation from normal mucosa to neoplasia/dysplasia were loss of crypt architecture, depletion of surface mucin, and increased mitosis as well as associated nuclear changes including crowding, stratification, elongation and hyperchromasia. In addition, the dysplastic red and green cells had an elevated level of CTNNB1 (β-catenin) and often this protein was localized to the nucleus confirming that cells in both lineages were neoplastic (Fig 1C–1F). Finally, red and green neoplastic clones often carried unique mutations in *Apc* (Table 2 and S4 Fig), further indicating that these clones are derived from different founders. Thus, this innovative mouse model is quite suitable for studying the clonal architecture of intestinal tumors. Moreover, an important advantage of this model is that the visualization of different lineages does not impede molecular analyses including immunohistochemistry and DNA sequencing.

**Table 1:** Clonal architecture of intestinal tumors from 20 mosaic mice euthanized at 150 days of age.

			Phenotype, N (%)		
Intestinal region	Pathological category	N	Heterotypic	Homotypic red	Homotypic green
Proximal SI	ND	165	0	165 (100%)	0
Distal SI & colon	Adenoma with LGD	63	32 (51%)	24 (38%)	7 (11%)

ND, not determined; SI, small intestine; LGD, low-grade dysplasia.

**Table 2:** Apc mutations in multi-ancestral tumors.

Tumor ID	Clone color	Nucleotide	Codon^a^	Base change	Amino acid change	Zygosity
6105 S4-A	Red	4109	1370	G > A	S > N	Heterozygous
	Green	2734	912	T > C	C > R	Heterozygous
6105 S4-C^b^	Red	2549	850	T > A	L > X	Heterozygous
	Green	2549	850	T > A	L > X	Homozygous
3866 CO-A	Red	2471	824	T > A	L > X	Heterozygous
	Green	2349	783	C > T	No change	Homozygous
	Green	2691	897	C > T	No change	Heterozygous
3704 CO-A	Red^c^	-	-	-	-	-
	Green	2506	836	A > G	R > G	Homozygous

^a^Mutations in Apc codons 747–953 and 1285–1513 were identified as described in Methods. The heterozygous Min mutation was detected in all tumor samples except 6105 S4-C green, in which the mutation was homozygous.

^b^Tumor 6105 S4-A was mostly red with only one small green clone as shown S4 Fig.

^c^The red tissue in tumor 3704 CO-A lacked the mutation at nucleotide 2506 found in the green tissue.

The percentage of multi-ancestral tumors in mosaics was similar to the percentage observed in earlier studies with aggregation chimeras (S2 Fig; 40–52% in mosaics versus 22–47% in aggregation chimeras). This finding most likely reflects the fact that the patchwork of red and green cells in the distal small intestine and colon of our mosaic mice was comparable to the patchwork of blue and white cells observed in aggregation chimeras in previous studies (S2 Fig). All 165 tumors in the proximal small intestine were homotypic red (Table 1), as the rat fatty acid binding protein 1 promoter is hardly expressed in this region of the intestinal tract (S3 Fig). This observation indicates that the expression of fluorescent proteins from the reporter is stable during tumorigenesis. Because tumorigenesis does not lead to activation of Cre or mutations in the reporter, heterotypic tumors can accurately be determined to have a multi-ancestral origin. This approach can generally be applied to study the clonal architecture of tumors in other tissues, requiring only the use of a different transgene expressing Cre recombinase.

**Fig 2:**
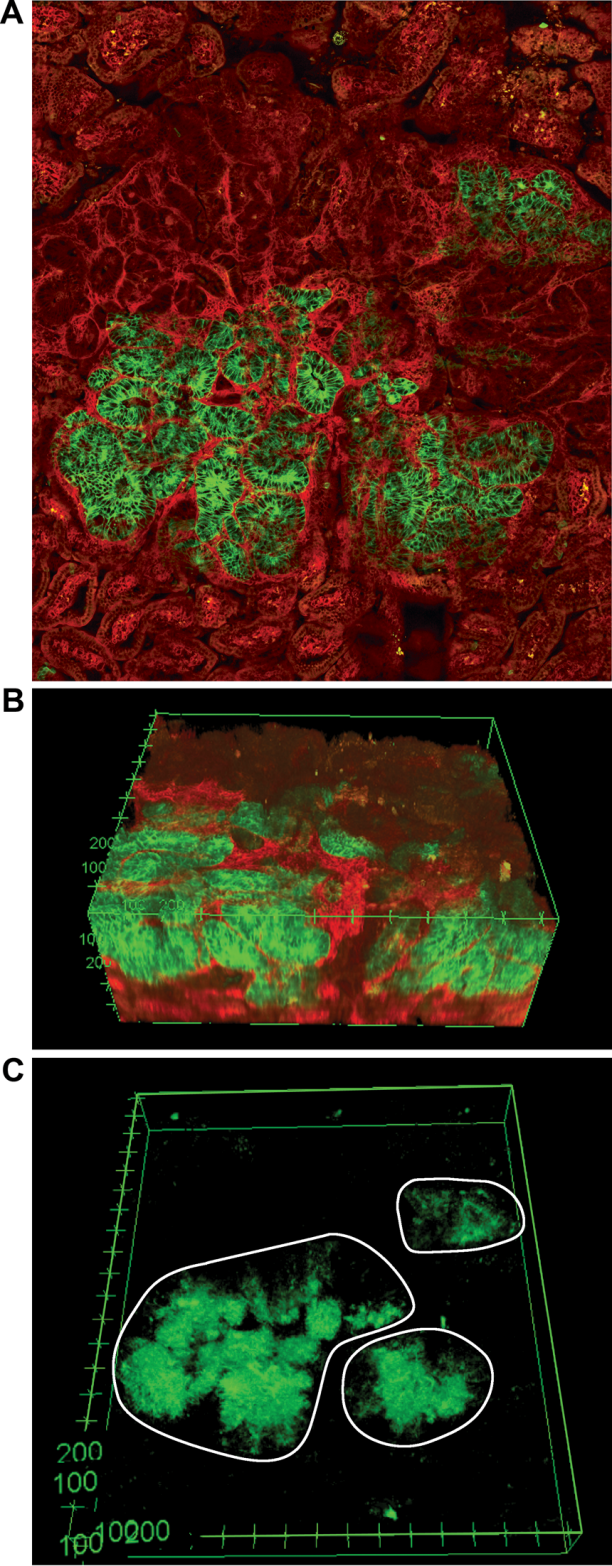
Multi-ancestral tumors appear to be derived from up to four founders. The expression of fluorescent proteins facilitates the use of multiphoton excitation imaging to reconstruct the tumor in three dimensions Reconstructions permit the exploration of structure beneath the surface of the tumors (A, surface; B, side view) without distortions and artifacts that occur when imaging multiple histological sections. Four distinct colored areas were evident in one tumor. Three discrete green areas were evident when the red was subtracted (C). These areas of green were separated by at least 100 microns, which is equivalent to the diameter of 1 to 2 crypts.

The multi-ancestral architecture of tumors was further confirmed via imaging. A total of five multi-ancestral adenomas were isolated, suspended in TDE as the mounting medium to reduce scattering from lipids, and imaged with a multiphoton microscope. For each adenoma, the collected images were stitched together to generate a three-dimensional reconstruction. These data indicate that some tumors might be more complex than simply biclonal; the number of discrete colored regions ranged from two to four (S2 Table). In one predominantly red adenoma, three separate green areas could be seen, indicating that this tumor could be derived from four founders if each clone arose from a unique initiating event (Fig 2). However, the preparation for imaging prevented us from being able to perform the sequencing studies that would allow us to determine if the separate green patches had different mutation profiles, which would provide further evidence of their origins.

**Fig 3:**
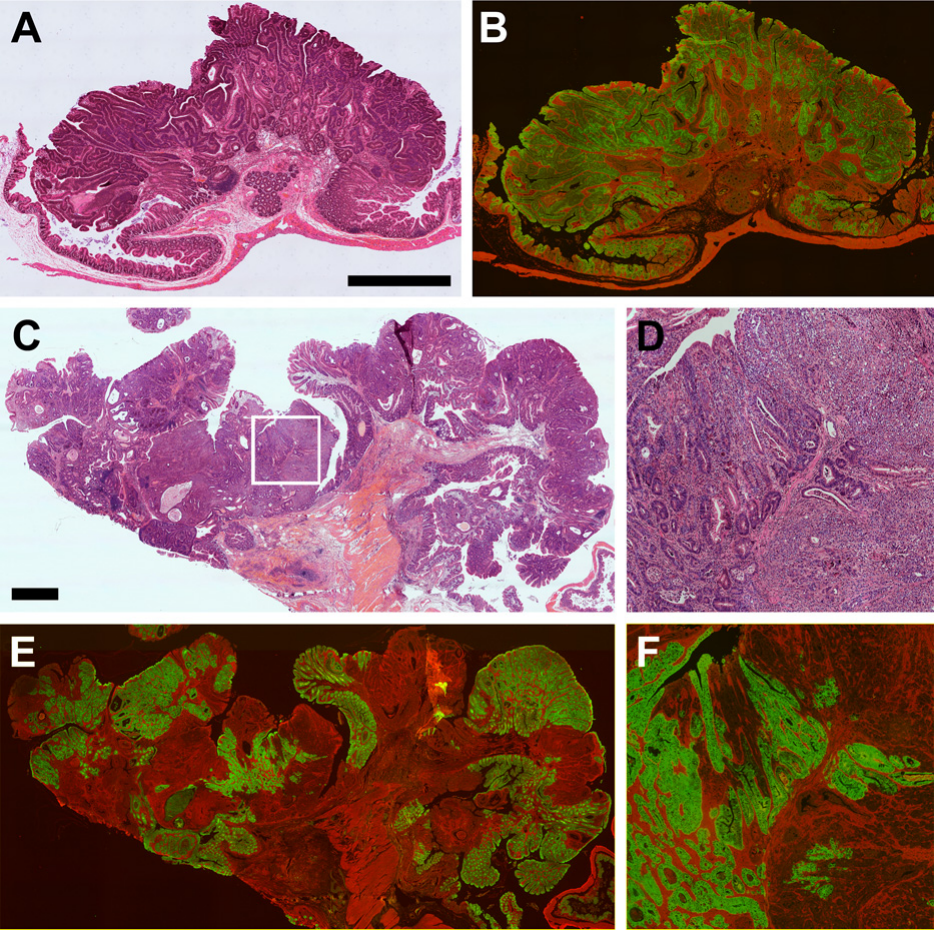
In mice that were allowed to age until moribund, some tumors were adenomas with high-grade dysplasia (A and B), whereas others were intramucosal carcinomas (C-F). The neoplastic cells identified on H&E stained sections (A, C and D) were a mixture of both red and green cells (B, E and F). Panels A and B are shown at the same magnification; size bar = 1mm. Panels C and E are same magnification; size bar = 1mm. Panels D and F are 4x enlargements of the area outlined in C.

The multi-ancestral architecture persists as tumors progress from benign to malignant states. A second cohort of mice was generated and allowed to age until moribund. The average age at death was 494 ± 93 days. The incidence of intestinal tumors was 100% with an average multiplicity of 15 ± 5, which was slightly higher than that observed in mice euthanized at 150 days of age (p = 0.04; Wilcoxon rank sum, two-sided). In the moribund mice, 16% of the tumors in the distal small intestine and colon were intramucosal carcinomas and 13% were invasive adenocarcinomas (Table 3). Similar to that observed in human colorectal lesions, intramucosal carcinomas exhibit neoplastic cell invasion into either the lamina propria or muscularis mucosa and can be associated with a desmoplastic response (Fig 3). Note that a previous study demonstrated that long-lived Min mice develop advanced disease even though the tumors exhibited little genetic instability [14]. For a lesion to be considered an invasive adenocarcinoma there had to be invasion of neoplastic cells beyond the muscularis mucosa at least into the submucosa. The neoplastic cells were often columnar in appearance with prominent nucleoli, had a cribriform architecture with back-to-back glands without intervening stroma, granular eosinophilic karyorrhectic debris present within the lumen of the gland, increased mitotic activity and associated with a desmoplastic response. A multi-ancestral architecture was observed in 62% of adenomas with low-grade dysplasia, 57% of adenomas with high-grade dysplasia, 43% of intramucosal carcinomas, and 68% of invasive adenocarcinomas (Table 3).

**Table 3:** Clonal architecture of intestinal tumors from the distal small intestine and colon of 31 mosaic mice euthanized when moribund.

		Phenotype, N (% of pathological category)		
Pathological category	N (% of total)	Heterotypic	Homotypic red	Homotypic green
Adenoma with LGD	65 (45%)	40 (62%)	23 (35%)	2 (3%)
Adenoma with HGD	37 (26%)	21 (57%)	15 (41%)	1 (3%)
Intramucosal carcinoma	23 (16%)	10 (43%)	13 (57%)	0
Invasive adenocarcinoma	19 (13%)	13 (68%)	6 (32%)	0
Total, N (%)	144 (100%)	84 (58%)	57 (40%)	3 (2%)

LGD, low-grade dysplasia; HGD, high-grade dysplasia.

Malignant cells invading the submucosa and muscularis propria can even be derived from different founders. A total of 15 invasive adenocarcinomas from 10 mice were analyzed. Simultaneous invasion of red and green neoplastic cells was observed in 13%. This observation indicates that interactions among clones may persist even as tumors begin to invade from the submucosa into the muscularis propria.

## Discussion

We generated a novel mouse model of colorectal cancer, performed validation studies to compare our findings with earlier experimental models, and then utilized the model to study the clonal architecture of malignant cancers in the intestine. The findings not only indicate that cancers are composed of cells derived from multiple founders but that cells from distinct origins can simultaneously invade into the colonic wall musculature. This discovery is contrary to the notion that all solid tumors are uni-ancestral and that a single subclone eventually acquires a selective advantage through epigenetic alterations or mutations and consequently metastasizes from the primary tumor to distant sites.

Our model was validated using several different approaches. The Fabp1-Cre transgene was selected because it is stably expressed in the distal small intestine, cecum, and colon during embryogenesis [24, 25] and before tumorigenesis occurs in the Min mouse. Consistent with this assertion, the crypts within the normal intestine were either wholly red in the absence of Cre expression or wholly green in the presence of Cre expression. Moreover, the pattern of mosaicism is similar in mice euthanized at 150 days of age and those killed when moribund, indicating that the pattern established early during embryogenesis is stable in adult mice (S5 Fig, S6 Fig, and S3 Table). If Cre was spontaneously activated in different cells, the intestine would progressively turn more green as mice age because green cells cannot be converted back into red. In both groups of mice, the proximal half of the intestinal tract was almost entirely red, whereas the distal half was a mixture of red and green with the colon being composed of 37.6% green cells in mice euthanized at 150 days and 38.1% green cells in mice euthanized when moribund (p = 0.46, t-test). All 165 tumors that formed in the proximal half of the small intestine and 24 of 63 tumors in the colon and distal half of the small intestine were homotypic red (Table 1). This observation indicates that Fabp1-Cre transgene is not aberrantly activated as a consequence of neoplastic transformation. Other tumors in the distal half of the intestinal tract were homotypic green or heterotypic. The percentage of heterotypic, or overtly multi-ancestral, tumors was similar to that observed in previous studies in which aggregation chimeras were employed (S2 Fig). The pattern of red and green neoplastic clones within a single tumor varies dramatically with red and green being highly intermingled in some tumors (Fig 1B). Such complex mixtures would be difficult to explain by the indeterminate expression of the Fabp1-Cre transgene within a single clone. Finally, within a heterotypic tumor, dysplastic crypts are wholly red or wholly green and distinct clones often carried a different *Apc* mutation (Table 2 and S4 Fig). Taken together, the data demonstrate that our new model is a powerful new tool for studying the clonal origin of intestinal tumors.

Early intestinal adenomas in mice and humans can have a multi-ancestral origin [2]. Many of the initial studies were performed utilizing aggregation chimeras in which two early embryos were fused together to generate a single animal. One embryo typically carried the Rosa26 lineage marker, whereas the other did not, so that cells from the two distinct lineages were distinguishable [26]. A significant number of intestinal adenomas were composed of cells from both lineages indicating those tumors were at least biclonal. Our new model enabled us to more closely examine the number of clones contributing to a single tumor because the tumors can be reconstructed through *ex vivo* imaging using a multiphoton microscope. The number of separate areas, which are likely to be discrete clones, contributing to a single tumor ranged from two to four (S2 Table; Fig 2). This observation is consistent with two other studies that recently demonstrated that cells from three distinct lineages could contribute to single intestinal tumor in the laboratory mouse [27, 28].

Intratumoral heterogeneity appears to arise at the earliest stage of tumorigenesis. We recently reported that multi-ancestral tumors form as a consequence of recruitment in which an initiated primogenitor facilitates the transformation of co-genitors [29]. Interactions among clones have been shown to occur at distances that encompass both first- and second-order neighboring crypts, about 18 crypts on average [4]. Fischer and colleagues recently demonstrated that a field of at least three Apc-deficient crypts is an important intermediate between loss of APC activity and adenoma formation [28]. This observation indicates that additional tumor founders are not mere passengers in the tumor, but might actually facilitate tumorigenesis.

Recruited co-genitors might already carry mutations in driver genes. Parsons and colleagues detected KRAS mutations in normal epithelial cells in the human colon [30]. These mutant cells might be more easily recruited to form a multi-ancestral tumor. Early intratumoral heterogeneity is consistent with the Big Bang Model of Tumorigenesis. Sottoriva and colleagues profiled 349 individual crypts from 15 colorectal tumors [31]. Based on their analysis, they proposed that tumors grow predominantly as a single expansion producing numerous intermixed subclones that are not subject to stringent selection with both clonal and subclonal alterations arising early. This work was further extended by Kang and colleagues who extensively analyzed a 6 cm colon tumor by measuring point mutations, chromosome copy numbers, and DNA passenger methylation [32]. They concluded that multiple related clones arise within the first few divisions. Thus, the recruitment of co-genitors with distinct mutation profiles to form a multi-ancestral tumor could further increase intratumoral heterogeneity. Additional studies are needed to further understand the number of founders present in a single tumor and the relationship among different clones.

Once a multi-ancestral tumor is established, our data indicate that multiple distinct clones persist as tumors progress. This finding complements that of one of the first studies to assess the clonal architecture of human colorectal cancers. In 1967, Beutler and colleagues analyzed a colorectal cancer and numerous liver metastases from an African American woman who was diagnosed in her twenties [33]. They found that the primary tumor and several metastases were derived from multiple progenitors. Thus, the clonal architecture might be a valuable predictor of response to therapeutic intervention, as heterogeneous tumors are more likely to be intrinsically resistant or acquire resistance.

Cells from distinct founders within a single tumor simultaneously invaded into the submucosa and muscularis propria. In our model, the cells have equal tumorigenic potential, although more homotypic red tumors were observed in the distal small intestine and colon than homotypic green tumors. Changes in tumor microenvironment that favor invasion could lead to cells from both lineages being present regardless of the invasive potential of any particular clone. However, if one clone gained a selective advantage over the other via mutation in any one of several key genes including *Kras*, *Pik3ca*, and *Tp53*, the dynamic might change such that only that clone invades. These possibilities could explain why not all metastases from the patient in the Beutler study were shown to be multi-ancestral. New mouse models coupled with state-of-the-art fluorescence endoscopy like that described in this study will enable us to more fully understand how clonal architecture can impact tumor growth, progression, and response to therapeutic intervention. We can generate mice with tumors that are composed of clones with different mutation profiles and monitor each clone over time to assess natural history or response to therapy. Such a study was not feasible with aggregation chimeras because the generation of mice with the proper genotype was challenging at best, e.g. the odds of fusing together embryos with desired alterations in two genes ranges from 1:16 to 1:256 depending on the breeding scheme and desired genotype.

Our new experimental system is a powerful tool to further our understanding of tumor origin, but it has limitations. First, the Fabp1-Cre transgene is activated early during embryogenesis and remains active throughout the life of the animal, so theoretically the expression pattern could change and cause a previously uni-ancestral tumor to become heterogeneous. However, we demonstrate that the pattern of expression does not change over time and that it is unaffected by tumorigenesis. Second, *Apc*^Min/+^ mice even with a F1 hybrid background have a shortened lifespan so tumors rarely metastasize to distant sites, therefore the clonal architecture of metastatic lesions can not be determined. Third, in *Apc*^Min/+^ mice, all cells in the entire organism carry one mutant allele of *Apc*. Such cells have altered properties including changes in proliferation, apoptosis, and migration [34]. We considered inducible systems including the Lgr5-CreERT2 transgene, which was developed and characterized by the laboratory of Dr. Hans Clevers [27]. However, with this system Cre is almost exclusively expressed in the small intestine and would leave us unable to assess the origins of colon tumors. The majority of human intestinal tumors form in the colon. Second, Lgr5-positive cells are thought to be at least a subpopulation of stem cells in the small intestine but it is unknown whether these cells are the only stem cells and the only cell type from which tumors arise. Third, inducing CRE expression in the intestine prior to the initiation of tumorigenesis in *Apc*^Min/+^ mice would be challenging as pregnant females or neonates would have to be treated with tamoxifen. In our experience, all (10/10) pregnant *Apc*^*Min/+*^ females with treated with tamoxifen lost their litters.

A widely accepted paradigm in the field of cancer biology has been that solid tumors are uni-ancestral and they transition from a benign to malignant states as subclones acquire epigenetic changes and mutations that drive progression. We demonstrated that early intestinal adenomas can be multi-ancestral arising from interactions among multiple founders. Our approach can be easily adapted to study the clonal architecture of tumors in other tissues, requiring only the tissue-specific, mosaic expression of Cre recombinase and a method to induce tumors. In addition, advances in sequencing are making the clonal analysis of human tumors quite feasible. Thus, a deeper understanding of clonal architecture is likely to impact cancer prevention, diagnosis, and treatment.

## Supporting Information

S1 Fig The color images of whole mounts were converted to black and white images so that the pattern of mosaicism could be characterized. An ideal model for studying polyclonality would have very small patch sizes; smaller patches enhance the ability to detect polyclonal tumors because the probability that founders arise from different marked lineages increases. Note that the larger patch size arising as a consequence of X-inactivation heavily biased the analysis of human tumors towards the conclusion that human colorectal tumors are monoclonal (Novelli et al., Science 1996;272:1187–90).
(EPS)

S2 Fig The percentage of multi-ancestral tumors is similar in different models. The ideal experimental system for studying the clonal origin and architecture of intestinal tumors would uniquely label each stem cell/crypt. With our system, patches of CRE-negative (tdTomato) and CRE-positive (EGFP) are relatively small and evenly distributed in the distal half of the small intestine and colon, which is conducive to identifying heterotypic, overtly multi-ancestral tumors if they exist. The distance between cells of different colors was calculated for mosaic mice in this study and aggregation chimeras in previous studies (Thliveris et al., Proc Natl Acad Sci U S A 2005;102:6960–5; Thliveris et al., Cancer Prev Res (Phila) 2011;4:916–23). The distance in the distal small intestine and colon from 150-day-old mice in this study was comparable to that observed in aggregation chimeras, indicating that the pattern of mosaicism was similar in the two different experimental platforms. Note that the small intestine was divided into four equal sections with Section 1 correlating to the duodenum, Section 2 plus 3 correlating to the jejunum, and Section 4 correlating to ileum. Since the pattern of mosaicism in the mice in this study varied along the length of the intestinal tract, the distances for section 4 and the colon were plotted separately. Note that the percentage of green cells was 12.0% in section 4 but 37.6% in the colon of mice euthanized at 150 days. By contrast, the pattern of mosaicism in aggregation chimeras from previous studies was quite consistent along the length of the intestinal tract, so the distances for sections 1, 2, 3, 4 and the colon were all plotted together. Power calculations considering the pattern of mosaicism indicate that we could detect multi-ancestral tumors in mosaics or aggregation chimeras if they were only 1 out of every 100 tumors.
(PDF)

S3 Fig The pattern of mosaicism varies along the length of the intestinal tract. The rat fatty acid binding protein promoter is expressed in very few cells in the duodenum and jejunum (sections 1–3) so these regions of the small intestine are primarily red, whereas it is expressed in many cells in the ileum (section 4) and colon so these regions are a mixture of red and green. The intestinal tract is shown with the duodenum at the top proceeding to the colon at the bottom. Size bar: 5mm.
(TIF)

S4 Fig Sequencing of *Apc* indicates that different clones within a single tumor are distinct. DNA was prepared from the red and green clones in the biclonal tumor that is shown in Fig 1 and a region of *Apc* was sequenced. The red clone carried a missense mutation at codon 4109 resulting in a change serine to asparagine, whereas the green clone carried a missense mutation at codon 912 resulting in a change from cysteine to arginine. Both clones carried the *Min* allele. Note that only a small region of exon 15 of the gene was sequenced.
(TIF)

S5 Fig The pattern of mosaicism is quite similar when comparing mice euthanized at 150 days to those euthanized when moribund. Colons are shown from mosaic mice euthanized at 150 days (A-E) and those euthanized when moribund (F-J). The percentage of green cells ranged from 28.6 to 44.6. Note large tumors are evident in the long-lived mosaic mice.
(PDF)

S6 Fig The pattern of mosaicism in the colon does not change over time. The percentage of cells expressing EGFP was plotted for mice euthanized at 150 days of age (n = 18) and those euthanized when moribund (n = 15). The average was 37.6% for 150-day-old mice and 38.1% for long-lived mice (p = 0.46, t-test).
(PDF)

S1 File Supporting Methods: Equipment and Settings. (PDF)

S1 Table Primers used for *Apc* sequencing. (PDF)

S2 Table 3D reconstruction of polyclonal adenomas revealed the number of discrete clones. (PDF)

S3 Table The pattern of mosaicism does not change with age. (PDF)
