# Supplementary figures and images for: Advanced Intestinal Cancers often Maintain a Multi-Ancestral Architecture
Source: PLoS One. 2016 Feb 26;11(2):e0150170. doi: 10.1371/journal.pone.0150170 (PMC4769224; doi:10.1371/journal.pone.0150170)

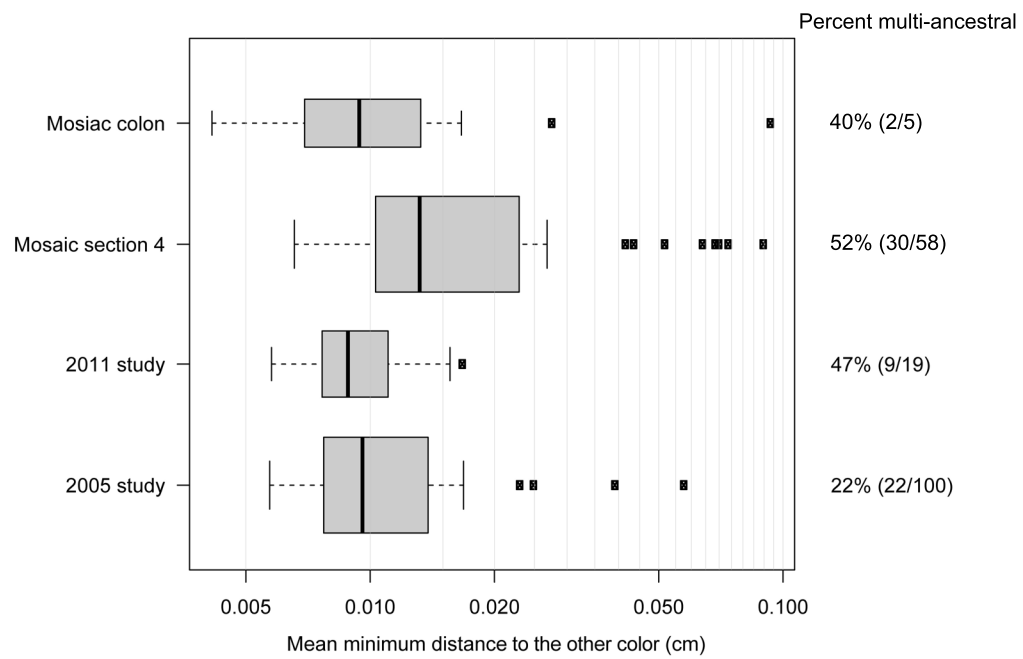

Supplement: S2 Fig — The ideal experimental system for studying the clonal origin and architecture of intestinal tumors would uniquely label each stem cell/crypt. With our system, patches of CRE-negative (tdTomato) and CRE-positive (EGFP) are relatively small and evenly distributed in the distal half of the small intestine and colon, which is conducive to identifying heterotypic, overtly multi-ancestral tumors if they exist. The distance between cells of different colors was calculated for mosaic mice in this study and aggregation chimeras in previous studies (Thliveris et al., Proc Natl Acad Sci U S A 2005;102:6960–5; Thliveris et al., Cancer Prev Res (Phila) 2011;4:916–23). The distance in the distal small intestine and colon from 150-day-old mice in this study was comparable to that observed in aggregation chimeras, indicating that the pattern of mosaicism was similar in the two different experimental platforms. Note that the small intestine was divided into four equal sections with Section 1 correlating to the duodenum, Section 2 plus 3 correlating to the jejunum, and Section 4 correlating to ileum. Since the pattern of mosaicism in the mice in this study varied along the length of the intestinal tract, the distances for section 4 and the colon were plotted separately. Note that the percentage of green cells was 12.0% in section 4 but 37.6% in the colon of mice euthanized at 150 days. By contrast, the pattern of mosaicism in aggregation chimeras from previous studies was quite consistent along the length of the intestinal tract, so the distances for sections 1, 2, 3, 4 and the colon were all plotted together. Power calculations considering the pattern of mosaicism indicate that we could detect multi-ancestral tumors in mosaics or aggregation chimeras if they were only 1 out of every 100 tumors. (PDF) [file pone.0150170.s002.pdf]

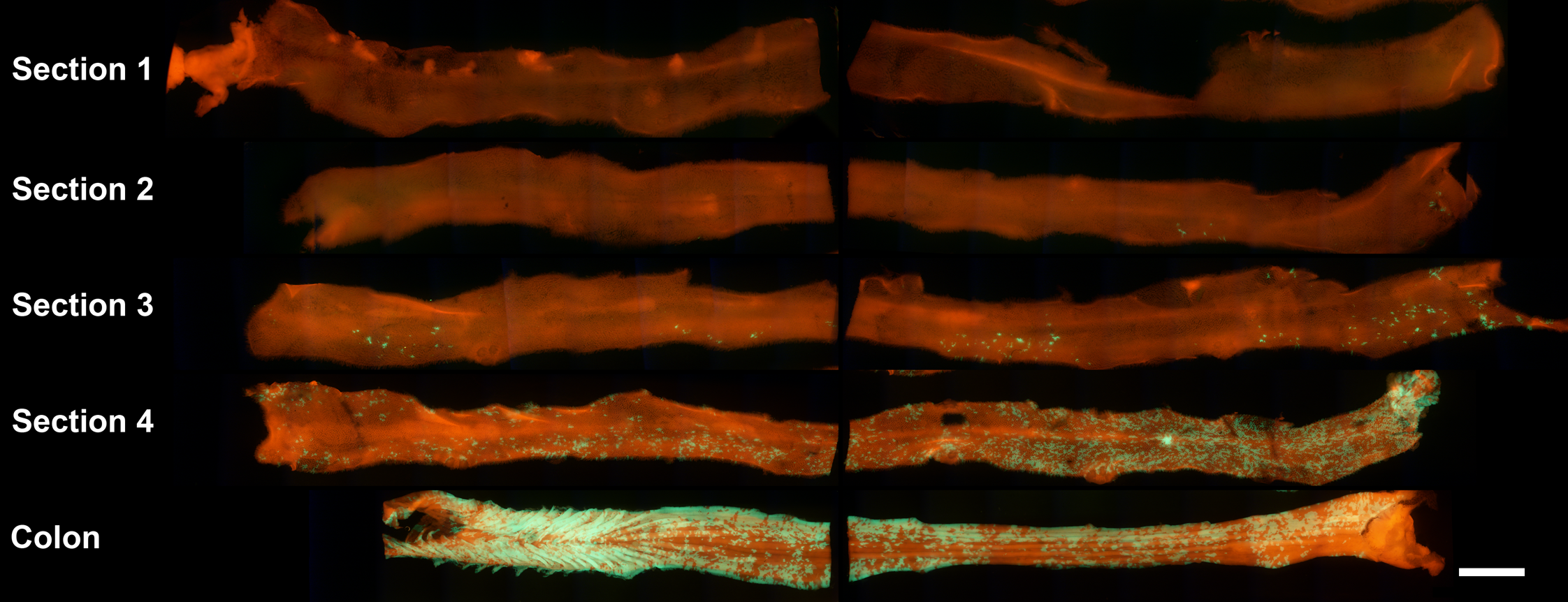

Supplement: S3 Fig — The rat fatty acid binding protein promoter is expressed in very few cells in the duodenum and jejunum (sections 1–3) so these regions of the small intestine are primarily red, whereas it is expressed in many cells in the ileum (section 4) and colon so these regions are a mixture of red and green. The intestinal tract is shown with the duodenum at the top proceeding to the colon at the bottom. Size bar: 5mm. (TIF) [file pone.0150170.s003.tif]

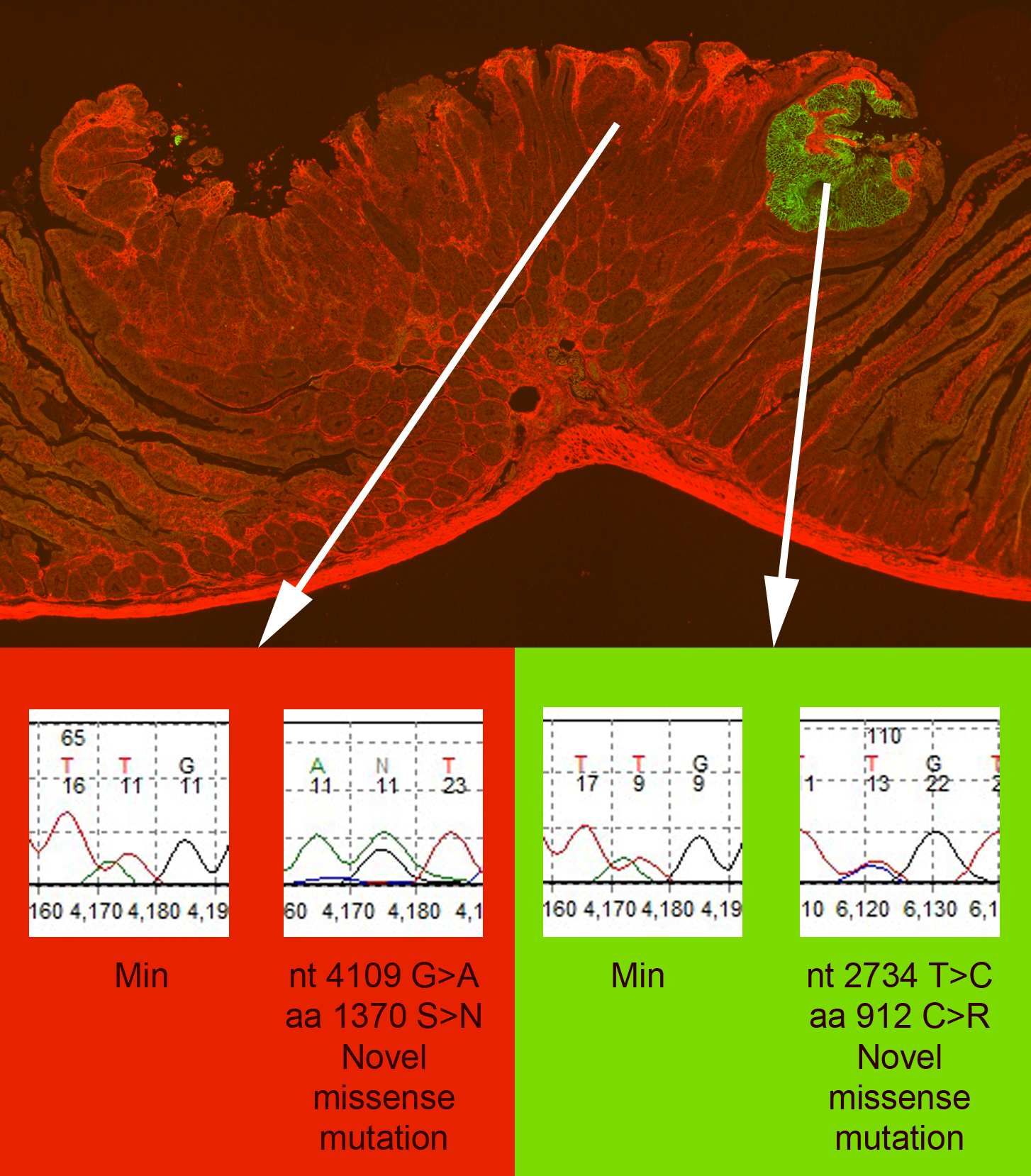

Supplement: S4 Fig — DNA was prepared from the red and green clones in the biclonal tumor that is shown in Fig 1 and a region of Apc was sequenced. The red clone carried a missense mutation at codon 4109 resulting in a change serine to asparagine, whereas the green clone carried a missense mutation at codon 912 resulting in a change from cysteine to arginine. Both clones carried the Min allele. Note that only a small region of exon 15 of the gene was sequenced. (TIF) [file pone.0150170.s004.tif]

150 days old

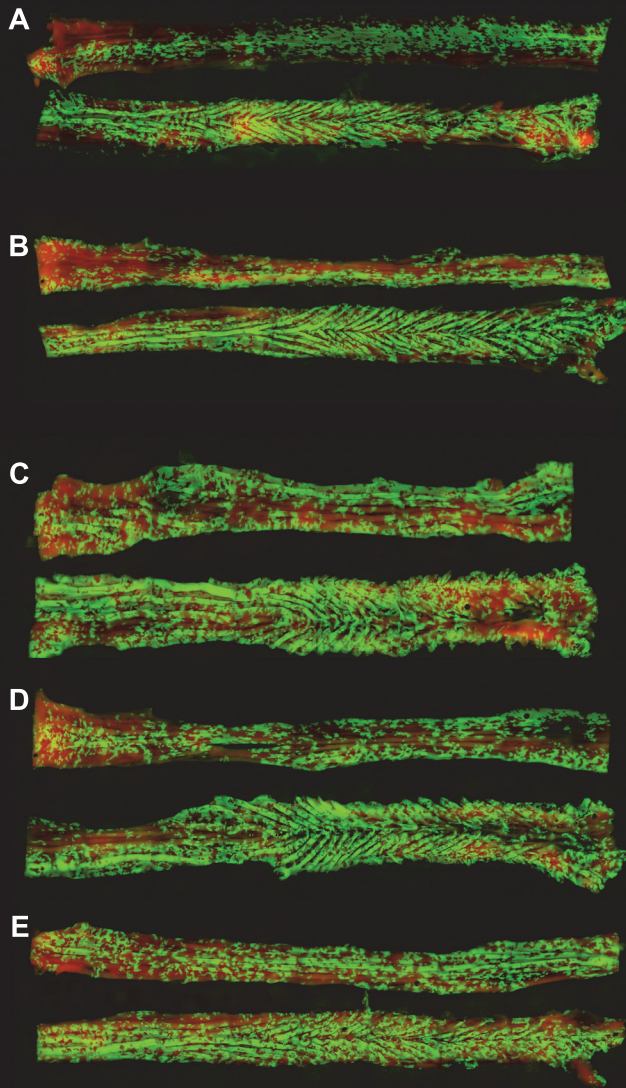

Long-lived

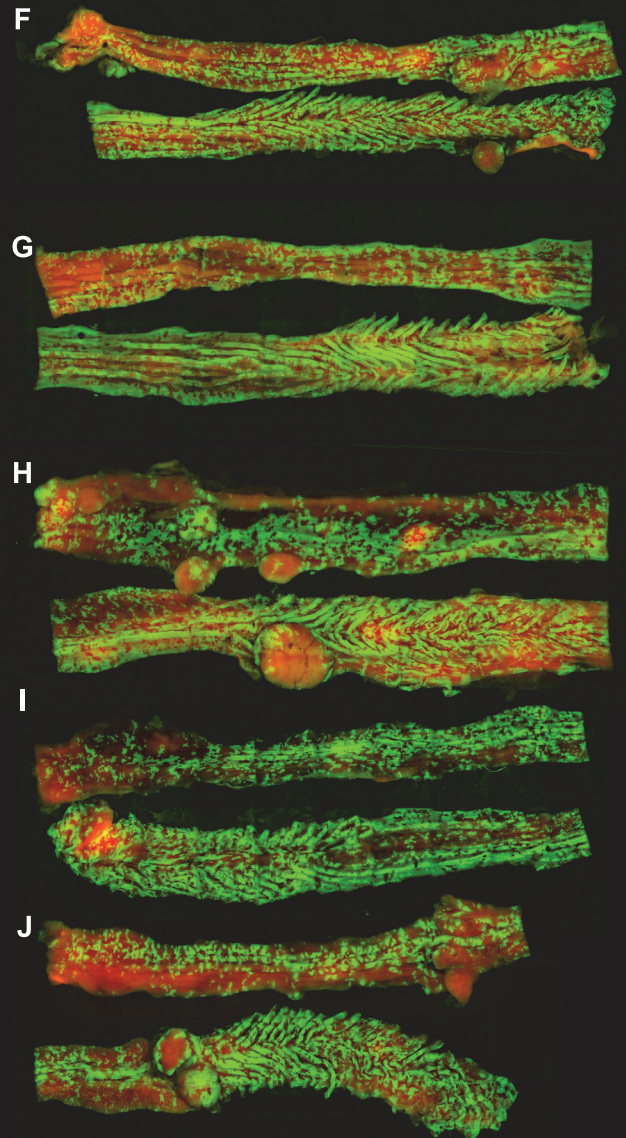

Supplement: S5 Fig — Colons are shown from mosaic mice euthanized at 150 days (A-E) and those euthanized when moribund (F-J). The percentage of green cells ranged from 28.6 to 44.6. Note large tumors are evident in the long-lived mosaic mice. (PDF) [file pone.0150170.s005.pdf]

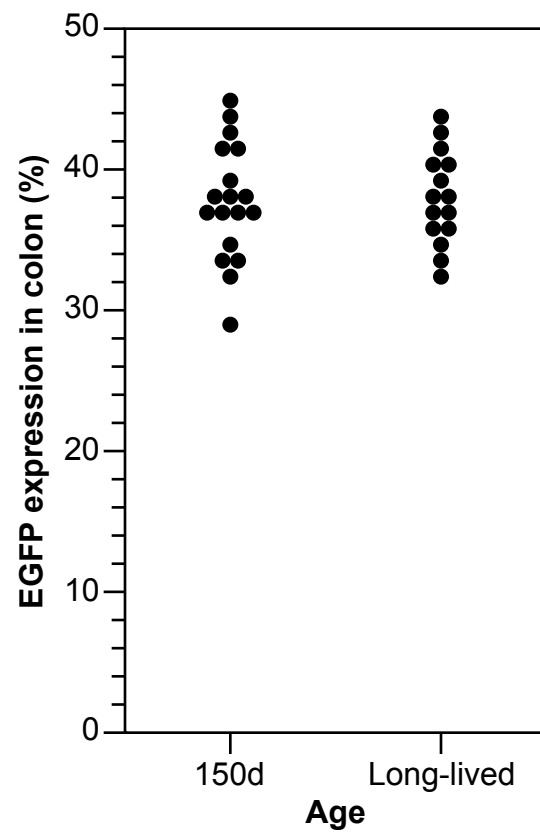

Supplement: S6 Fig — The percentage of cells expressing EGFP was plotted for mice euthanized at 150 days of age (n = 18) and those euthanized when moribund (n = 15). The average was 37.6% for 150-day-old mice and 38.1% for long-lived mice (p = 0.46, t-test). (PDF) [file pone.0150170.s006.pdf]
