# Supplementary material for: Advanced Intestinal Cancers often Maintain a Multi-Ancestral Architecture
Source: PLoS One. 2016 Feb 26;11(2):e0150170. doi: 10.1371/journal.pone.0150170 (PMC4769224; doi:10.1371/journal.pone.0150170)
Supplement: S1 File — (PDF) [file pone.0150170.s007.pdf]

Supplementary Methods

Equipment and Settings

Whole mount and histological tissue sections were imaged on one of three microscopes:

1. Zeiss AxioPlan II
   Software: AxioVision 4.8.2 with the Mosaix package
   Camera: Zeiss AxioCam MR3
   Fluorescent excitation source: HBO 103 W/2 mercury vapor short-arc lamp (product# 000000-1007-978-000)
   Objective lenses: Plan-Neofluar 1.25x NA 0.04 (item #440300-0000-000-000), Plan-Neofluar 2.5x NA 0.075 (item #440310-0000-000-000), Plan-Neofluar 10x NA 0.30 (item #440330-0000-000-000), Plan-Apochromat 20x NA 0.75 (item #440649-0000-000-000)
   Filters sets used: Zeiss filter set 1 - EX 365/12nm, EM LP 397nm (item # 488001-9901-000), Zeiss filter set 10 - EX 470/40nm, EM 540/50nm (item # 488010-9901-000), Zeiss filter set 31 - EX 565/30nm, EM 620/60nm (item# 000000-1031-350)

2. Nikon Eclipse TI-S
   Software: NIS-Elements: Advanced Research with the 6D acquisition module
   Camera: Nikon Digital Sight DS-Ri1 (product #MQA16050)
   Fluorescent excitation source: C-HGFI Intensilight mercury illuminator (product#MBF72655)
   Objective lenses: CFI Plan Achromat UW 2X NA 0.06 WD 7.5MM (product #MRL00022), CFI Plan Fluor 4X NA 0.13 WD 17.2MM, Eco-Glass (product #MRH00041), CFI Plan Fluor 10X NA 0.3 WD 16MM, Eco-Glass (product #MRH00101), CFI Plan Fluor 20X NA 0.5 WD 2.1MM, Spring-Loaded Eco-Glass (product #MRH00201)
   Filters sets: C-FL UV-2E/C DAPI Filter Block DM-400, EX 360/40, EM 460/50 (product # 96310), C-FL B-2E/C FITC Filter Block DM 505, EX 480/30, EM 535/40 (product # 96311), C-FL G-E/C TRITC Filter Block DM 565, EX 540/25, BA 620/60 (product # 96312)

3. Prairie Technologies Ultima IV
   Software: PrairieView v.4
   Detector: Hamamatsu Multi alkali Photo Multiplier Tubes
   Fluorescent excitation source: Spectra-Physics Insight DeepSee Ultrafast Laser
   Objective lenses: Zeiss Plan-Apochromat 20x NA 1.0
   Filters sets used: Chroma 525/50nm and 620/60nm filters and a T545lp dichroic mirror

Acquisition settings for images used in figures are as follows:

Figure 1B: All images were taken with the Zeiss microscope using the 2.5x objective lens and filter sets 10 and 31 for the EGFP and tdTomato, respectively. The camera was set to 32-bit RGB color with a resolution of 4.98µm/pixel and linear look up tables. Exposure settings were as follows (left to right):

tdTomato 153 ms; EGFP 522 ms.

tdTomato 366 ms; EGFP 1046 ms.

tdTomato 175 ms; EGFP 540 ms.

tdTomato 160 ms; EGFP 274 ms.

tdTomato 425 ms; EGFP 800 ms.

Figure 1C-F: All images were taken with the Nikon microscope using linear look up tables and the following objectives and settings:

Panel C – Imaged using the 10x objective lens with the camera set to 8-bit RGB color with no filters at a resolution of 0.92µm/pixel.

Panel D – Imaged using the 20x objective with the camera set to 8-bit RGB color with no filters at a resolution of 0.46µm/pixel.

Panels E and F – Imaged using the 20x objective lens with the FITC and TRITC filter blocks for EGFP and tdTomato, respectively. The camera was set to 8-bit emulated monochrome with a resolution of 0.46µm/pixel. Exposure times were 600ms for EGFP and 1s for tdTomato. The gain was set to 1x for EGFP and 3.4x for tdTomato.

Figure 2: All panels were created from the same original image set taken on the Prairie Technologies Ultima IV microscope using the 20x objective lens, Spectra-Physics Mai Tai DeepSee set to a wavelength of 1000nm for excitation, and emission was filtered using the T545lp dichroic mirror with Chroma filters 525/50nm and 620/60nm for EGFP and tdTomato, respectively. The images were taken at a resolution of 0.94µm/pixel in the X and Y planes and with a step of 2µm/image in the Z plane.

Figure 3: Images for Panels A and B were taken with the Nikon microscope using the 10x objective lens at a resolution of 0.92µm/pixel. Panels C and E were taken with the Nikon microscope using the 4x objective lens at a resolution of 0.37µm/pixel. Panels D and F were taken with the Nikon microscope using the 20x objective lens at a resolution of 0.46µm/pixel. All panels use linear look up tables.

Panels A, C, and D – The camera was set to 8-bit RGB color with no filters.

Panels B, E, and F – The camera was set to 8-bit emulated monochrome using the FITC and TRITC filter blocks for EGFP and tdTomato, respectively. Exposure times were 800ms for EGFP and 3s for tdTomato. The gain was set to 1x for EGFP and 3.4x for tdTomato.

Supplementary Figure S3 Fig: All sections were imaged with the Nikon microscope using the 2x objective lens with the FITC and TRITC filter blocks for EGFP and tdTomato, respectively. The camera was set to 8-bit RGB color with a 2x2 bin and a resolution of 9.15µm/pixel. Linear look up tables were used. Exposure times and gains were as follows:

Section 1 (top) – tdTomato 30 ms, 1.4x gain; EGFP 20 ms, 1.4x gain.

Section 2 – tdTomato 30 ms, 1.4x gain; EGFP 20 ms, 1.4x gain.

Section 3 – tdTomato 40 ms, 1.4x gain; EGFP 20 ms, 1.4x gain.

Section 4 – tdTomato 80 ms, 1.4x gain; EGFP 20 ms, 1.4x gain.

Colon (bottom) – tdTomato 80 ms, 1.4x gain; EGFP 20 ms, 1.4x gain.

Supplementary Figure S4: Image used is Figure 1 panel E.
