# Supplementary material for: Advanced Intestinal Cancers often Maintain a Multi-Ancestral Architecture
Source: PLoS One. 2016 Feb 26;11(2):e0150170. doi: 10.1371/journal.pone.0150170 (PMC4769224; doi:10.1371/journal.pone.0150170)
Supplement: S1 Table — (PDF) [file pone.0150170.s008.pdf]

S1 Table. Primers used for *Apc* sequencing

Primer Name	Forward primer		Reverse primer		Product Size (bp)
	Sequence	Melting temperature (^{o}C)	Sequence	Melting temperature (^{o}C)	
ApcEx15a-1	CACGTTAGGAAACAGAAAGC	58.3	AAAAGCATAGTCACCATAAA	54.7	147
ApcEx15a-2	CGGAGTAAGCAGAGACACAA	60.7	TCTAAACTTCCCCTTGAGGA	59.3	170
ApcEx15a-3	CATGACTGTTCTTTCACCAT	57.4	TCGTTTTGATGAGGTTCCTG	59.3	175
ApcEx15a-4	GCCTCAGTGCTTACCATCCA	63.2	CTGTCGTCTGCCACACAATG	62.5	181
ApcEx15a-5	TCCCAGGACGACAGAAGTTC	62.5	TGGCATAAGGCATAGAGCAT	60.7	160
ApcEx15b-1	GGTGCAGTTCATTATCATCA	57.3	CTCTAGCATTCTGGGACACT	60.1	177
ApcEx15b-2	AATGATGTAACTCGGTCAGC	59.2	GGGGGACTTTTGGGTGTCTG	63.9	194
ApcEx15b-3	GCCAAGTCTCCCTCCAAAAG	61.9	GCCACTCACCATTCCACTAC	61.6	177
ApcEx15b-4	TTTGAGAGTCGCTCCATTGC	61.9	ACTTTACTTTTTGGCACCTC	57.9	185
ApcEx15b-5	ACCTCCTCCACAGACAGTGC	64.8	GCTGGAGGAACAAGAAAACC	60.4	196
