# Supplementary material for: Advanced Intestinal Cancers often Maintain a Multi-Ancestral Architecture
Source: PLoS One. 2016 Feb 26;11(2):e0150170. doi: 10.1371/journal.pone.0150170 (PMC4769224; doi:10.1371/journal.pone.0150170)
Supplement: S2 Table — (PDF) [file pone.0150170.s009.pdf]

S2 Table. **3D reconstruction of polyclonal adenomas revealed the number of discrete clones.**

		N of distinct regions	
Tumor ID	Intestinal region	Red	Green
6105C	Distal small intestine	1	3
3859E	Distal small intestine	1	2
3862C	Distal small intestine	1	2
3862E	Distal small intestine	1	1
4000A	Colon	1	1
