# Supplementary material for: Advanced Intestinal Cancers often Maintain a Multi-Ancestral Architecture
Source: PLoS One. 2016 Feb 26;11(2):e0150170. doi: 10.1371/journal.pone.0150170 (PMC4769224; doi:10.1371/journal.pone.0150170)
Supplement: S3 Table — (PDF) [file pone.0150170.s010.pdf]

S3 Table. The pattern of mosaicism does not change with age. SI, small intestine.

	Percentage of tissue expressing EGFP, mean ± SD		
Intestinal section	150 day old mice (n = 18)	Long-lived mice (n = 15)	p value (t-test)
SI section 1	0.0 ± 0.0	0.0 ± 0.0	0.26
SI section 2	0.0 ± 0.0	0.1 ± 0.1	0.07
SI section 3	0.4 ± 0.6	0.5 ± 0.6	0.68
SI section 4	12.0 ± 2.6	11.5 ± 1.4	0.33
Colon	37.6 ± 4.1	38.1 ± 3.3	0.46
